# Single-dose SARS-CoV-2 vaccinations with either BNT162b2 or AZD1222 induce disparate Th1 responses and IgA production

**DOI:** 10.1186/s12916-022-02240-4

**Published:** 2022-01-19

**Authors:** Michael Müller, Johann Volzke, Behnam Subin, Silke Müller, Martina Sombetzki, Emil C. Reisinger, Brigitte Müller-Hilke

**Affiliations:** 1 Core Facility for Cell Sorting and Cell Analysis, Rostock University Medical Center, Rostock, Germany; 2 Department of Cardiology, Rostock University Medical Center, Rostock, Germany; 3 Institute of Pharmacology and Toxicology, Rostock University Medical Center, Rostock, Germany; 4 Division of Tropical Medicine and Infectious Diseases, Center of Internal Medicine II, Rostock University Medical Center, Rostock, Germany

**Keywords:** SARS-CoV-2, Vaccination, AZD1222, BNT162b2, COVID-19, Type 1 helper T cells, Cytotoxic T cells

## Abstract

**Background:**

While vaccination programs against the severe acute respiratory syndrome virus 2 (SARS-CoV-2) are globally ongoing, disparate strategies for the deployment of spike antigen show varying effectiveness.

**Methods:**

In order to explore this phenomenon, we sought to compare the early immune responses against AZD1222 and BNT162b2. SARS-CoV-2 seronegative participants received a single dose of either vaccine and were analyzed for immune cell, effector T cell, and antibody dynamics.

**Results:**

AZD1222 induced transient leukopenia and major changes among innate and adaptive subpopulations. Both vaccines induced spike protein-specific effector T cells which were dominated by type 1 helper T cell responses following AZD1222 vaccination. A significant reduction of anti-inflammatory T cells upon re-stimulation was also restricted to AZD1222 vaccinees. While IgM and IgG were the dominant isotypes elicited by AZD1222, BNT162b2 led to a significant production of IgG and IgA.

**Conclusions:**

Our results suggest that the strategy for spike protein delivery impacts on how and to what extent immune priming against the main SARS-CoV-2 antigen proceeds.

**Supplementary Information:**

The online version contains supplementary material available at 10.1186/s12916-022-02240-4.

## Background

The highly transmissible severe acute respiratory syndrome coronavirus 2 (SARS-CoV-2), which initially emerged in December 2019, has led to an unprecedented pandemic that caused over 4 million casualties [1, 2]. The prevailing occurrence of coronavirus disease 2019 (COVID-19) and its dramatic hazard for global health and economy has since spiked the rapid development of several vaccines. These collectively aim at the production of antibodies that will neutralize the binding of the viral spike glycoprotein to its angiotensin-converting enzyme 2 (ACE2) receptor and thereby prevent cellular entry and subsequent infection [3–5].

The urgent need to develop safe and efficient vaccines led to the deployment of various strategies, some of which were well established and others, like adenoviral vectors or mRNA, were novel. Among the early vaccines authorized by the European Medicines Agency (EMA) were the first-generation adenoviral vector AZD1222 that utilizes the simian dsDNA adenovirus ChAdOx1 as a vector for antigen delivery [6]. The first vaccine authorized by EMA was the nucleic acid-based BNT162b2, a spike protein encoding N1-methyl-pseudouridine (m1Ψ) nucleoside-modified mRNA enveloped by lipid nanoparticles [7, 8].

Complete vaccination with either of the vaccines, which includes two doses at varying intervals, was shown to efficiently protect from symptomatic COVID-19 [9, 10]. Although early data hint at similar efficiencies after a single dose of either vaccine, booster immunization with BNT162b2 achieved somewhat higher rates of thwarting viral breakthrough [9–13]. With the emergence of SARS-CoV-2 variants that accumulate mutations in the spike glycoprotein [14–16], the discrepancies between both vaccines grew even more pronounced with BNT162b2 leading to superior protection against the 1.351 (β) and 1.617.2 (δ) variants [15, 17–19].

We were curious about the molecular and cellular immune modules capable of mediating superior neutralization of SARS-CoV-2 and therefore aimed at exploring the immediate immune dynamics after a single dose of either AZD1222 or BNT162b2. To that extent, we investigated the proportions of peripheral leukocytes among innate and adaptive compartments over the first 3 weeks after immunization. To investigate the adaptive immune response in more detail, we surveyed the development of spike-protein-specific plasma immunoglobulins as well as the re-activation and cytokine production of spike-specific effector T cells.

## Results

### Immune responses to AZD1222 and BNT162b2 differ quantitatively and qualitatively

A total of 40 participants were recruited from the local coordination center for clinical studies. Twenty of these participants were vaccinated with AZD1222 (Vaxzevria/Astrazeneca) and BNT162b2 (Comirnaty/Biontech), respectively. Two participants from each group had to be excluded retrospectively. One individual from the AZD1222 group was excluded because this subject was tested positive for antibodies at baseline and the others withdrew their consent for unknown reasons. Blood samples were obtained by venipuncture on the day of vaccination (day 0) and 2, 6, 13, and 20 days later. Among all participants, 28 were available for all five consecutive venipunctures, five for four, two for three, and one for two venipunctures. Table 1 lists the demographic data of all participants, showing an even distribution of sex and comparable age ranges between both vaccination groups.

**Table 1:** Demographics of study participants

	AZD1222 (*n* = 18)	BNT162b2 (*n* = 18)	*p*-value
Sex [male/female]	9/9	6/12	0.5236*
Age [mean ± SD]	36.7 ± 11.8	39.2 ± 11.5	0.4998^#^

*Resulting from Fisher’s exact test, ^#^resulting from unpaired *t*-test

**Fig. 1:**
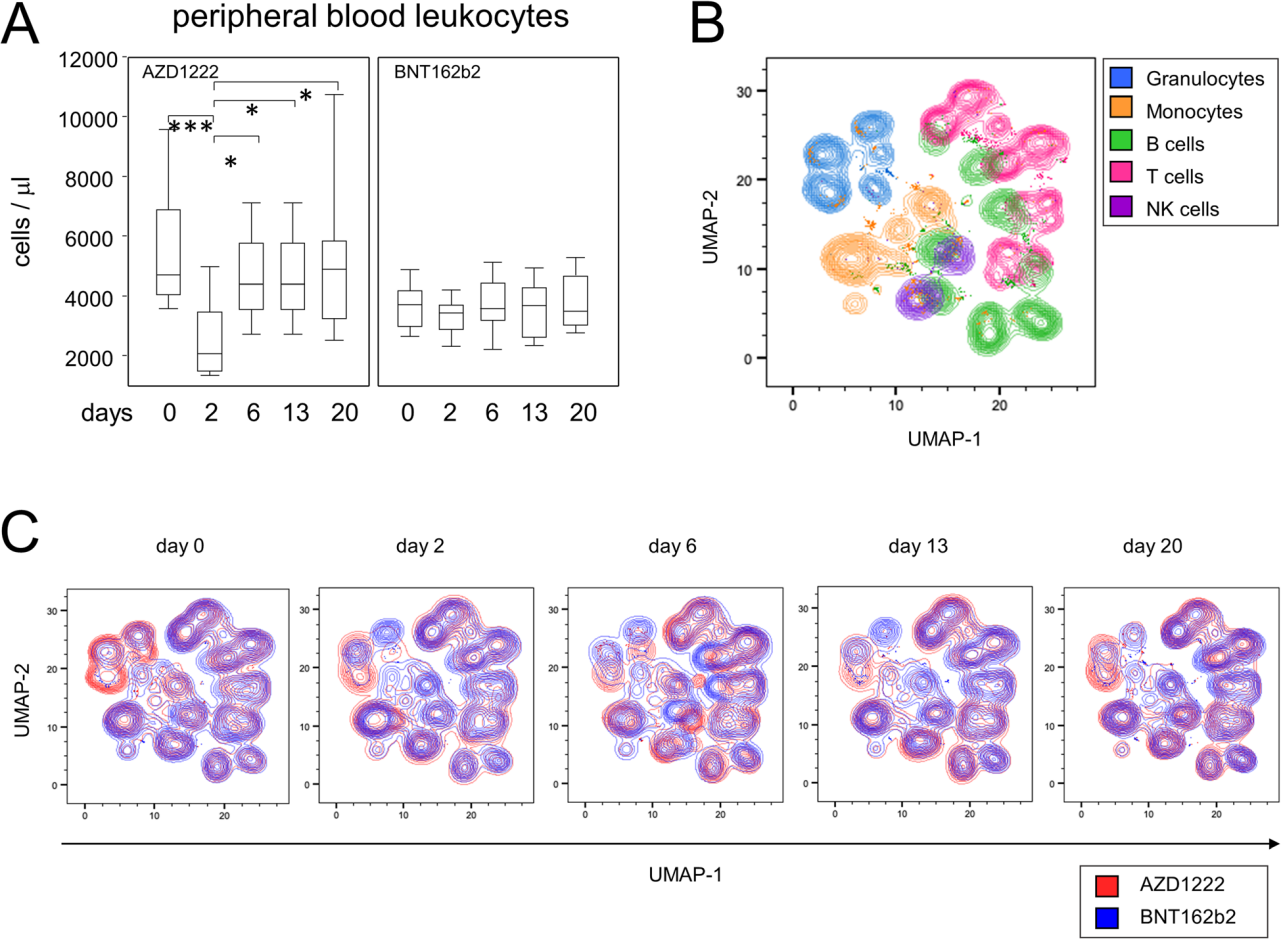
Vaccination with AZD1222 induced a transient reduction of peripheral leukocytes and displacements of major immune cell populations. **A** Leukocyte counts in the peripheral blood after vaccination with AZD1222 (*n* = 13, left panel) and BNT162b2 (*n* = 15, right panel). *p*-values resulting from multiple group comparisons were 0.0005 (AZD1222/Kruskal-Wallis and Dunn’s multiple comparisons tests) and 0.5306 (BNT162b2/one-way ANOVA), respectively. **p < 0.01, ***p < 0.001. **B** UMAP of surface antigen expression and clustering of major immune cell populations for all time points after vaccination. **C** UMAPs on immune cell compositions for each time point

In order to delineate the early immune cell responses to both vaccines, we performed 24-dimensional flow cytometry at each time point. Remarkably, by examining major immune cell compositions, we found in samples that were available for all consecutive time points a significant reduction (2.3-fold) in peripheral leukocytes on day 2 after vaccination with AZD1222 (Fig. 1A). This leukopenia resulted from significant reductions in granulocytes and B-lymphocytes as well as CD4- and CD8-positive T cells (see Additional file 1: Table S1). When compared to baseline, leukocyte counts were still slightly reduced on day 6 yet back to normal on days 13 and 20. In contrast, vaccination with BNT162b2 did not result in any significant fluctuations among immune cell quantities (Additional file 1: Table S2).

In order to survey qualitative alterations in the immune responses to either vaccine, we performed dimension reductions on our multiparametric data set by using the embedding algorithm “uniform manifold approximation and projection” (UMAP). Figure 1B summarizes all data from all time points and shows the topological distribution of immune cell subpopulations based on surface antigen expression patterns. Upon uncompressing the various time points, the overlay of both vaccine responses illustrates ample variation for the abundance of granulocyte, monocyte, and B cell subpopulations primarily after administration of AZD1222, while differences regarding T- and NK cell subpopulations were less prominent for both vaccination regimens (Fig. 1C). Taken together, our data show that vaccination of SARS-CoV-2 seronegative participants with AZD1222—unlike BNT162b2—resulted in a transient reduction of peripheral leukocytes and led to alterations in immune cell compositions.

### AZD1222 vaccination led to significant alterations among innate immune cell proportions

**Fig. 2:**
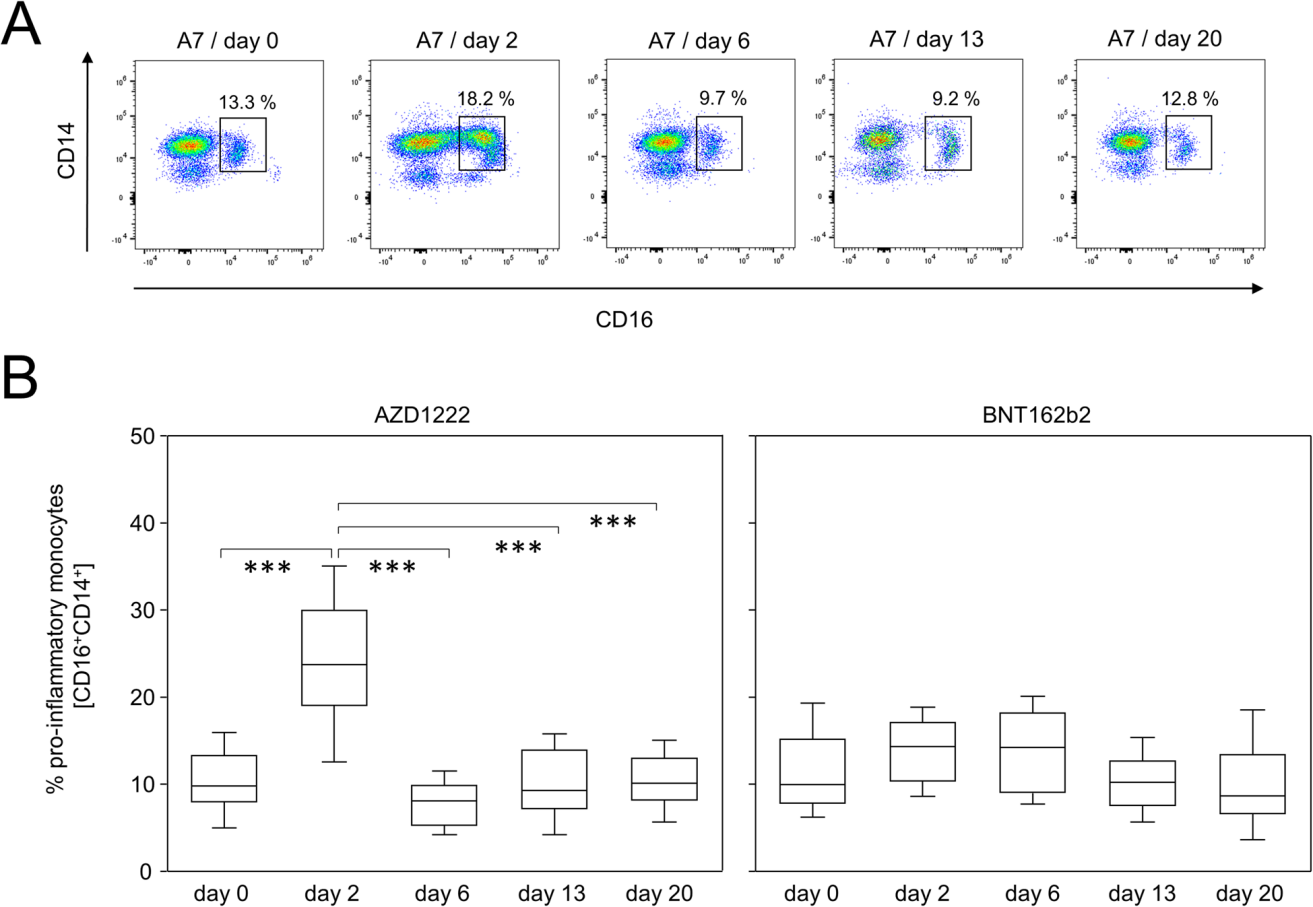
AZD1222 vaccination induced the enrichment of pro-inflammatory monocytes. **A** Pseudocolor plots for the expression of CD14 and CD16 on monocytes are representative for the AZD1222 vaccination group. **B** Proportions of CD16^+^CD14^+^ pro-inflammatory monocytes after vaccination with AZD1222 (*n* = 18, left panel) or BNT162b2 (*n* = 18, right panel). All FACS analyses were on gated live monocytes. Asterisks indicate significant differences between time points. *p*-values resulting from one-way ANOVA and Tukey-Kramer multiple comparisons tests were < 0.0001 for AZD1222 and 0.0146 for BNT162b2 analyses. ***p < 0.001

To further substantiate the time lines of early immune events following AZD1222 and BNT162b2 vaccination, we analyzed the major immune cell populations in more detail. Live monocytes were identified by their sideward scatter properties before alterations of CD14 and CD16 expression patterns were analyzed at various time points. Figure 2A illustrates the time line for one participant receiving AZD1222. Figure 2B documents a transient yet statistically significant increase in CD14^+^CD16^+^ pro-inflammatory monocytes on day 2 for the AZD1222 group (*p* < 0.0001). In contrast, there was no alteration among the proportions of pro-inflammatory monocytes following vaccination with BNT162b2 (Fig. 2B).

Likewise, there were significant changes among granulocyte subpopulations and these were restricted to AZD1222-vaccinated subjects only (Additional file 1: Figs. S1 and S2). In detail, CD177^–^CD11b^+^ among CD14^+^CD16^–^ granulocytes were significantly elevated on days 2 and 13 following vaccination (Additional file 1: Fig. S1). By day 20, this subpopulation was still increased to some extent however, due to high variance, the difference to baseline did not reach statistical significance. Interestingly, dynamics of CD177^–^CD11b^–^ among CD14^+^CD16^+^ granulocytes followed an opposing trend with proportions being decreased on days 2 and 13 before returning to baseline by day 20 after vaccination (Additional file 1: Fig. S2). In summary, this expression data show that vaccination with BNT162b2 had almost no impact on the peripheral innate immune compartment, whereas vaccination with AZD1222 led to marked alterations in the compositions of granulocyte and monocyte subpopulations.

### Changes among adaptive immune cell populations were most prominent after the AZD1222 vaccination

**Fig. 3:**
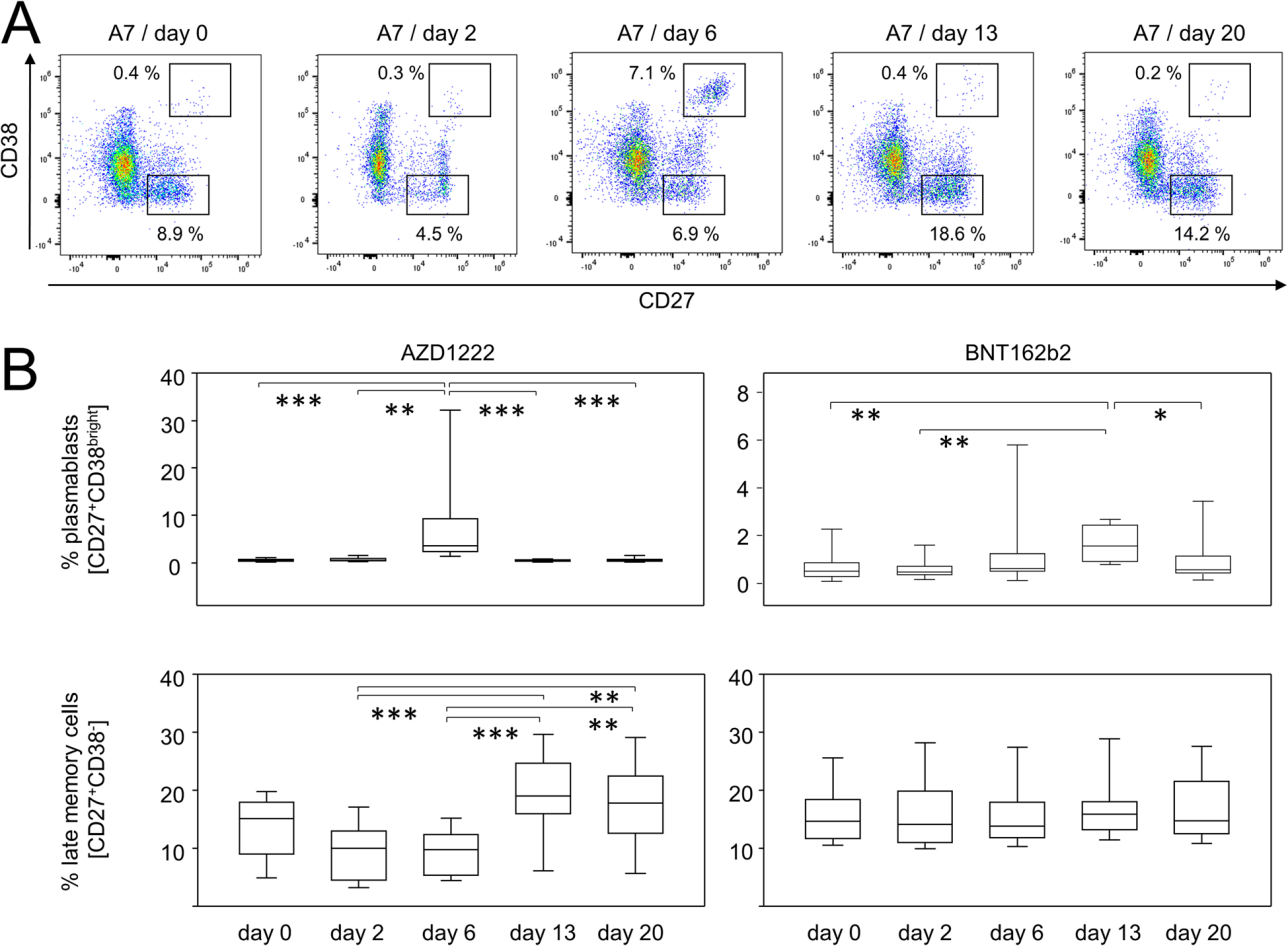
Vaccination with AZD1222 and BNT162b2 induced significant yet transient alterations among peripheral plasmablasts. AZD1222 also induced a significant increase in late memory B cells on days 13 and 20. **A** Pseudocolor plots for the expression of CD27 and CD38 on B cells are representative for the AZD1222 vaccination group. **B** Proportions of CD27^+^CD38^bright^ plasmablasts (top) and CD27^+^CD38^–^ late memory B cells (bottom) after vaccination with AZD1222 (*n* = 18, left) or BNT162b2 (*n* = 18, right). All FACS analyses were on CD19^+^CD45RA^+^ B cells. *p*-values resulting from one-way ANOVAs were < 0.0001 for both AZD1222 analyses. *p*-values resulting from Kruskal-Wallis and Dunn’s multiple comparisons tests were 0.0008 for the comparison of plasmablast and 0.6998 for the comparison of late memory B cells for the BNT162b2 analyses. *p < 0.05, **p < 0.01, ***p < 0.0001

In order to characterize the response of adaptive immune cells following vaccination, we next investigated the proportions of B- and T-lymphocyte subpopulations. Figure 3A shows representative data of CD19^+^CD45RA^+^ B cells and illustrates for a participant vaccinated with AZD1222 a shift of subpopulations expressing CD27 and CD38, respectively. While CD27^+^CD38^bright^ plasmablasts were significantly enriched on day 6 following vaccination with AZD1222 and reached a median of 3.08%, a plasmablast peak after BNT162b2 vaccination was detectable on day 13, yet reached a median of 1.57% only (Fig. 3B, upper panels). The increase in plasmablasts after the AZD1222 vaccination was flanked by an increase in CD27^+^CD38^–^ late memory B cells on days 13 and 20 (Fig. 3B, lower panels). No such alterations were observed after the BNT162b2 vaccination.

Likewise, alterations among T cell subpopulations were restricted to AZD1222 vaccinees only (Additional file 1: Figs. S3 and S4). In detail, we detected a shift in the expression patterns of CD27 and CD38 on CD8^+^ cells especially 2 days after AZD1222 vaccination (Additional file 1: Fig. S3A). This translated into a significant enrichment of CD27^–^CD38^+^ terminally differentiated cytotoxic T cells for this group (Additional file 1: Fig. S3B). Additional file 1: Fig. S4A exemplifies for CD4^+^ cells a change in the expression patterns for CD27 and CD127 on day 20 after AZD1222 administration. We thus discovered that CD4^+^CD127^–^CD27^+^ effector memory T cells re-expressing RA (TEMRA) were enriched towards the end of the observation period (Additional file 1: Fig. S4B). Taken together, we here demonstrated that significant changes among subpopulations of B- and T-lymphocytes were observed after vaccination with AZD1222 only.

### AZD1222 and BNT164b2 vaccinations led to significantly different helper and cytotoxic T cell polarizations

So far we have shown that a single dose of AZD1222 was capable of significantly modifying immune cell compositions. However, we assumed that both vaccines would on a small scale induce a specific cellular immune response towards the SARS-CoV-2 spike protein, which would become detectable upon re-stimulation with the antigen. We therefore used both, recombinant spike protein and BNT162b2 vaccine, and aimed to investigate cytokine profiles as well as the expression of inducible activation markers. In case of the recombinant spike protein, we expected it to be taken up by antigen-presenting cells (APCs). Upon processing the protein in lysosomes, respective peptides would predominantly become displayed on human leukocyte antigen (HLA) class II molecules and thus would be ready to activate spike protein-specific T helper cells. By employing the mRNA vaccine, we anticipated its cellular uptake, translation into protein and then both, secretion for uptake by APCs and class II presentation as well as processing the protein for presentation via HLA class I molecules and thereby re-stimulating cytotoxic T cells [20].

In order to establish a working protocol, we used peripheral blood mononuclear cells (PBMCs) from fully vaccinated or COVID-19 convalescent blood donors and investigated the expression of the activation marker CD137 on unstimulated cells compared to cells challenged with either the recombinant spike protein or BNT162b2. Indeed, we found a significant activation of CD4^+^ but not CD8^+^ cells after providing the recombinant spike protein (Additional file 1: Fig. S5). In contrast, stimulation with the BNT162b2 vaccine led to a significant increase of CD137 expressing cells among both, CD4^+^ and CD8^+^ lymphocytes (Additional file 1: Fig. S5).

**Fig. 4:**
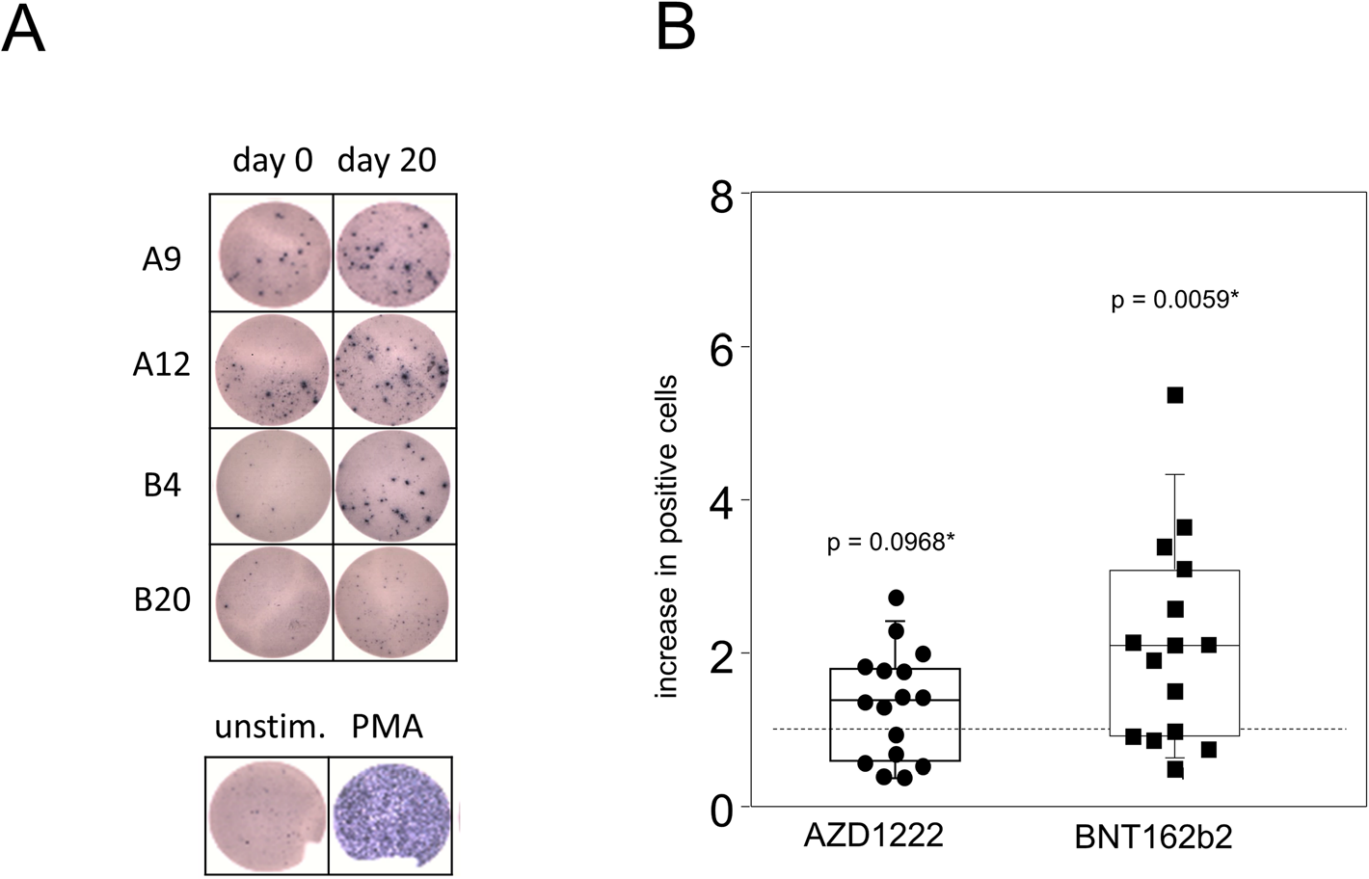
Spike-specific IFNγ-producing lymphocytes were significantly increased after vaccination with BNT162b2. PBMCs were isolated at the day of vaccination (day 0) and 20 days later. Cells were then stimulated with recombinant SARS-CoV-2 spike protein for 24 h. Subsequently, an ELISPOT assay for the detection of IFNγ-producing cells was performed. **A** Representative shots of spot-forming cells from individuals vaccinated with either AZD1222 (A9 and A12) or BNT162b2 (B4 and B20). PBMCs were stimulated with PMA as a positive control (bottom). **B** The counts of spot-forming cells from day 20 were normalized to counts from day 0. Data show the relative increase in IFNγ^+^ cells after vaccination (*n* = 16 for AZD1222 and *n* = 15 for BNT162b2). *p*-values result from one-sample *t*-tests assessing the differences of group means to the hypothetical value of 1

In a first approach, we used a classical enzyme-linked immune absorbent spot (ELISPOT) assay in combination with recombinant spike protein to confirm for both vaccination regimes increasing amounts of interferon (IFN)γ-secreting cells on day 20 compared to day 0. While Fig. 4A presents individual examples of ELISPOTs, Fig. 4B summarizes all results and indeed shows a significant increase in IFNγ-positive cells after BNT162b2 vaccination (*p* = 0.0059). There was also a trend towards increased IFNγ-positive cells after AZD1222 vaccination; however, this did not reach statistical significance (*p* = 0.0968).

**Fig. 5:**
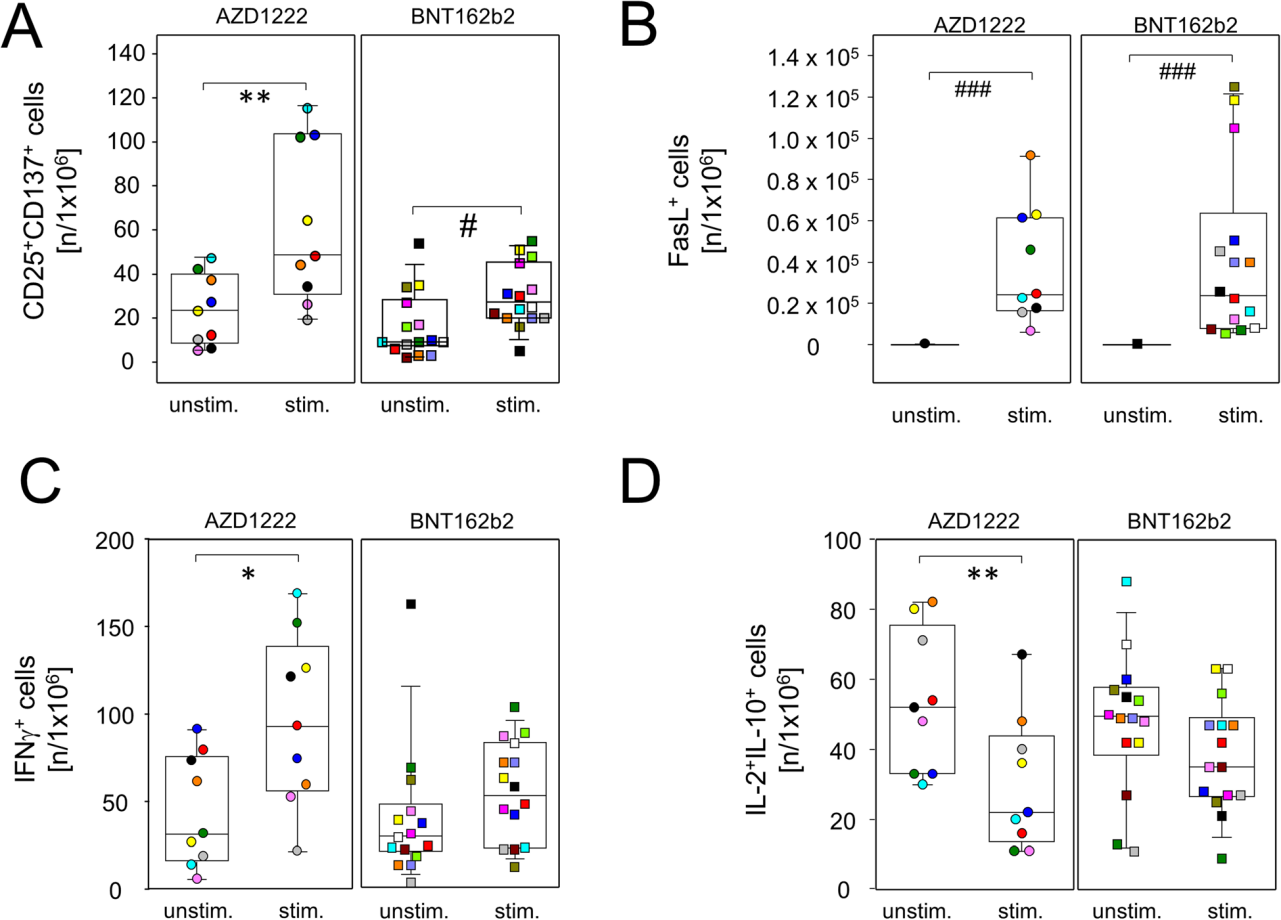
Vaccination with single doses of AZD1222 or BNT162b2 induced the expansion of cytotoxic effector T cells. PBMCs were isolated on day 20 after vaccination and stimulated with (stim.) or without (unstim.) spike protein-encoding mRNA (BNT162b2) for 24 h and analyzed by flow cytometry for the expression of inducible activation markers and intracellularly trapped cytokines among CD8^+^ cells. Sample sizes were *n* = 9 for AZD1222 and *n* = 15 for BNT162b2, respectively. Paired samples are illustrated by color coding. In vitro re-stimulation significantly increased CD25^+^CD137^+^ (**A**) and FasL expressing cells in both vaccination groups (**B**). INFγ–producers were increased (**C**) and IL-2 and IL-10 co-expressing cells were significantly reduced among AZD1222 vaccinees only (**D**). *p < 0.05, **p < 0.01 resulting from paired *t*-tests. ^#^*p* < 0.05, ^###^*p* < 0.001 resulting from Wilcoxon signed rank tests for matched pairs

We next sought to differentiate the AZD1222- and BNT162b2-induced adaptive cellular immune responses in more detail. We were interested in the activation profiles of both CD4- and CD8-positive T cells and therefore chose BNT162b2 for re-stimulation over the spike protein because the latter failed to induce a response by CD8^+^ T cells (Additional file 1: Fig. S5). Therefore, we cultured day 20 PBMCs from both vaccination groups in the presence or absence of BNT162b2 and surveyed activation and cytokine profiles by flow cytometry after Brefeldin A-capture of secretory proteins. Figure 5A shows that following both vaccination regimes, re-challenge with spike mRNA led to significantly increased amounts of CD8^+^CD137^+^ T cell that also expressed CD25 (IL-2Rα), suggesting the differentiation to an effector phenotype. In addition, both vaccines facilitated the expansion of inducible spike-specific cytotoxic effector T cells as demonstrated by significantly increased percentages of FasL^+^CD8^+^ cells after in vitro re-stimulation (Fig. 5B). We further detected a significant increase in IFNγ-producing CD8^+^ T cells from AZD1222- but not from BNT162b2-vaccinated donors (*p* = 0.0325 vs 0.1514, Fig. 5C). Finally, re-stimulation with spike mRNA decreased interleukin (IL-)2^+^IL-10^+^ co-expressing regulatory CD8^+^ cells for both AZD1222 and BNT162b2, the latter short of reaching statistical significance (*p* = 0.0058 vs 0.0768, Fig. 5D).

**Fig. 6:**
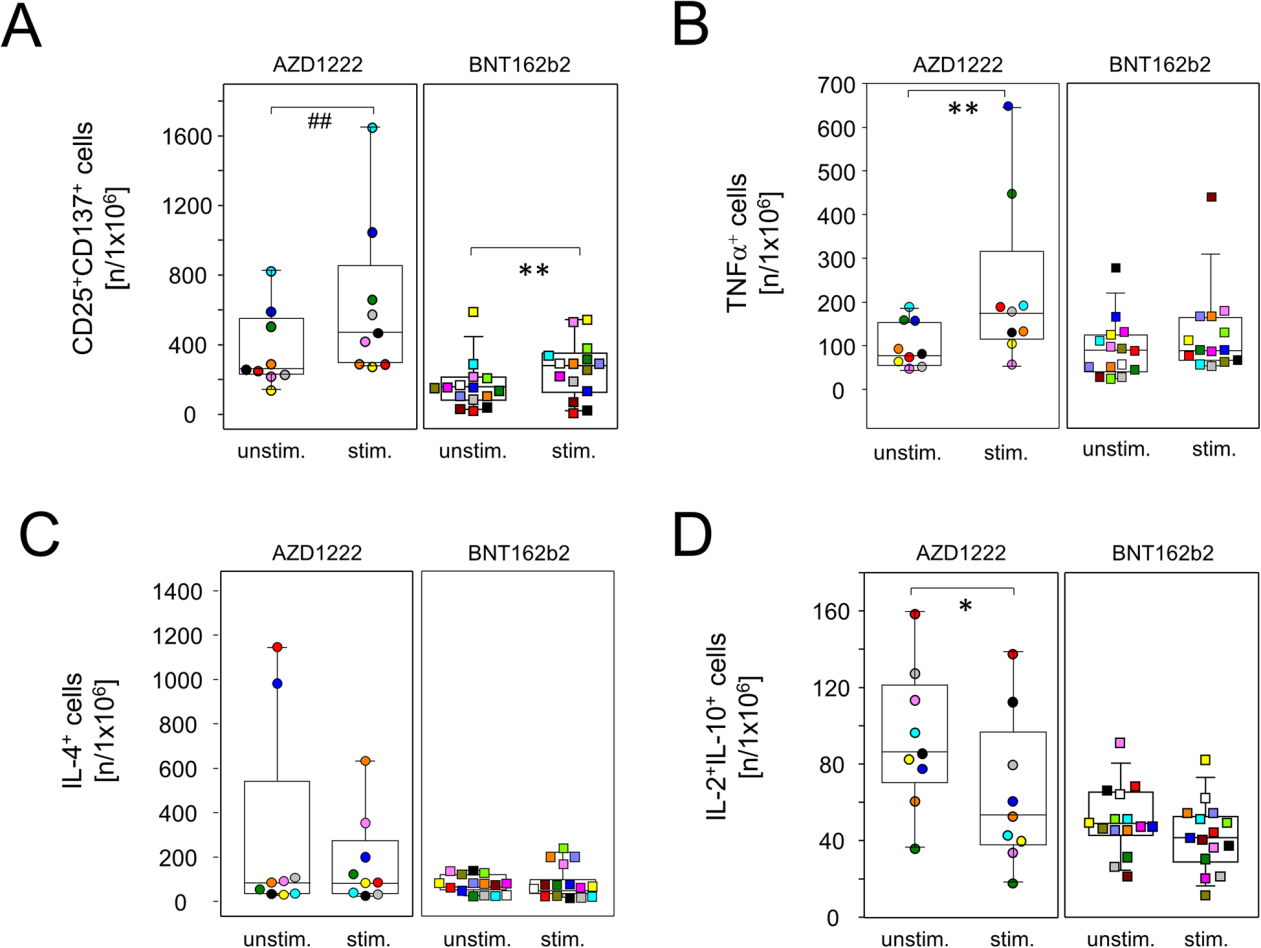
AZD1222 vaccination supported the induction of type 1 helper T cells. PBMCs were isolated on day 20 after vaccination and stimulated with (stim.) or without (unstim.) spike protein encoding mRNA (BNT162b2) for 24 h and analyzed by flow cytometry for the expression of inducible activation markers and intracellularly trapped cytokines among CD4^+^ cells. Sample sizes were *n* = 9 for AZD1222 and *n* = 15 for BNT162b2, respectively. Paired samples are illustrated by color coding. In vitro re-stimulation significantly increased CD25^+^CD137^+^ cells in both vaccination groups (**A**) and TNFα-producing cells in the AZD1222 vaccinated group, only **B**. **C** Neither vaccination allowed for the re-stimulation of IL-4-producing cells. **D** IL-2 and IL-10 co-expressing cells were significantly reduced among AZD1222 vaccinees only. *p < 0.05, **p < 0.01, resulting from paired *t*-test. ^##^*p* < 0.01, resulting from Wilcoxon signed rank test for matched pairs

Significantly increased amounts of T helper cells with an effector phenotype (CD4^+^CD25^+^CD137^+^) were also detected for both vaccine regimens after in vitro re-stimulation (Fig. 6A). In contrast, an increase in pro-inflammatory helper T cells producing tumor necrosis factor (TNF)α was restricted to re-stimulated cultures from day 20 AZD1222 donors (Fig. 6B). Of note, neither vaccination induced the expansion of spike-specific type 2 helper T cells as demonstrated by a lack of inducible IL-4 production by CD4^+^ cells after re-stimulation (Fig. 6C). Similar to regulatory CD8^+^ cells and again restricted to the AZD1222 group, re-stimulation with spike mRNA statistically significantly reduced anti-inflammatory IL-2 and IL-10 co-production in a subset of CD4^+^ cells (Fig. 6D).

In summary, these results demonstrate that single doses of either AZD1222 or BNT162b2 induced the polarization of spike-specific CD4^+^ and CD8^+^ effector T cells. More pronounced changes were again observed after vaccination with AZD1222 that included reduced proportions of IL-2 and IL-10 co-expressing CD4^+^ and CD8^+^ cells alike, combined with increased IFNγ-producing CD8^+^ and TNFα-producing CD4^+^ cells.

### BNT162b2 vaccination led to significantly more spike protein-specific plasma IgG and IgA

**Fig. 7:**
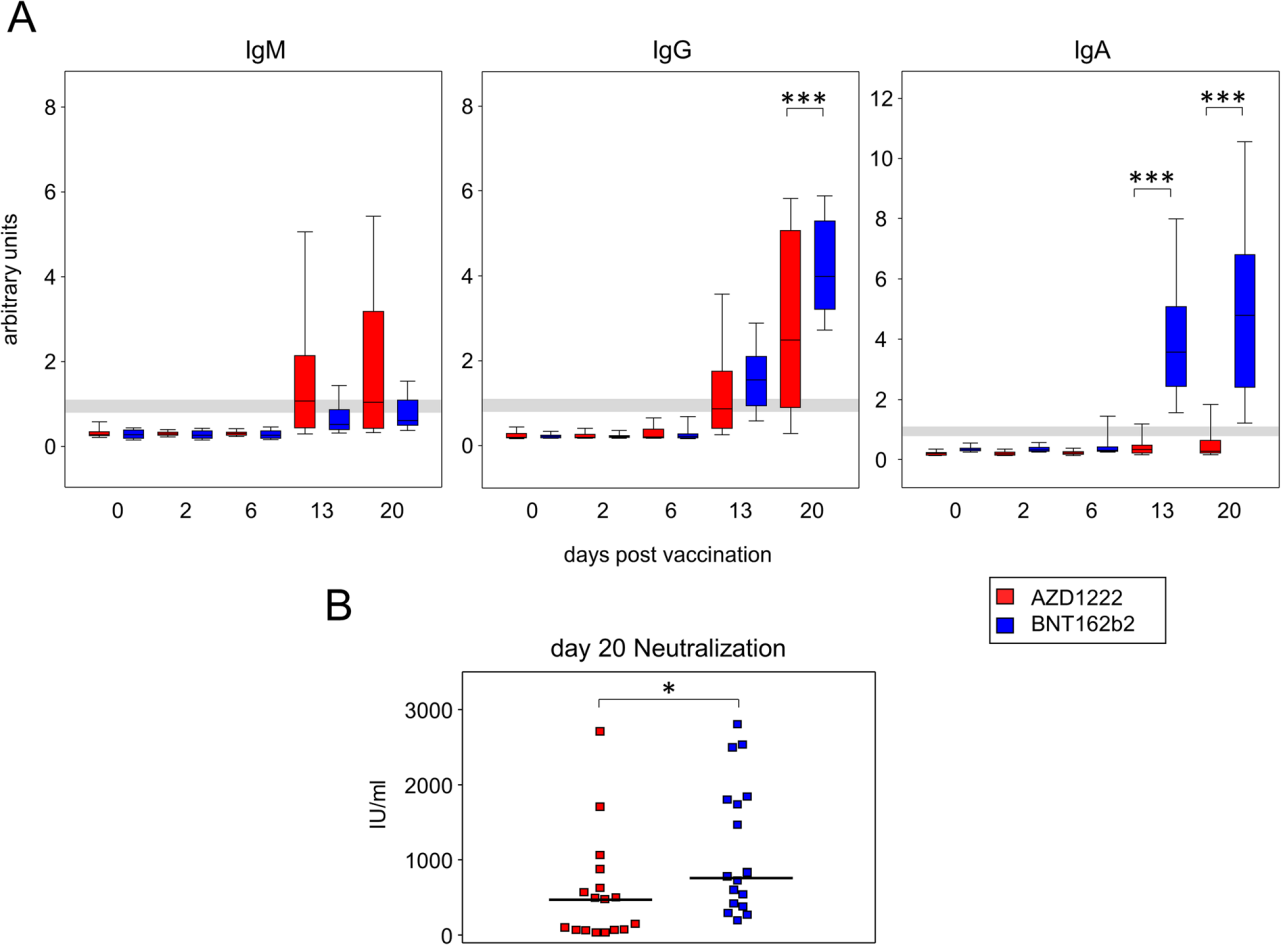
A single dose of BNT162b2 induced significantly higher amounts of spike protein binding IgG, IgA, and receptor binding domain neutralizing antibodies than AZD1222. **A** SARS-CoV-2 spike binding antibodies were detected by ELISA and absorbance readouts were normalized to calibrator values to obtain arbitrary units. Gray lines depict the positive response threshold within a range of 0.8 to 1.1 arbitrary units. Sample sizes were *n* = 18 for AZD1222 and *n* = 18 for BNT162b2, respectively. *p*-values resulting from one-way ANOVA and Tukey-Kramer multiple comparisons tests were < 0.0001 for all three isotype analyses. **B** Neutralizing antibodies were determined by the surrogate virus competitive ELISA. Absorbance readouts from BNT162b2 (*n* = 17) and AZD1222 (*n* = 17) were calibrated with WHO standards to obtain international units (IU)/mL. *p < 0.05 resulting from Mann-Whitney *U* test and ***p < 0.001 resulting from Dunn’s test

Even though both vaccines led to significant adaptive immune activation, alterations to inducible effector functions followed distinct patterns for AZD1222 and BNT162b2, respectively. We therefore sought to investigate whether these differences resulted in the production of diverse collections of immunoglobulin isotypes. To that extent, spike protein-specific IgM, IgG, and IgA were assessed for all time points via an enzyme-linked immunosorbent assay (ELISA). As shown in Fig. 7A, both vaccines induced the production of detectable amounts of antibodies as early as day 13. However, significant differences emerged between AZD1222 and BNT162b2 vaccination concerning the distribution and amounts of spike-specific antibody isotypes. In detail, AZD1222 predominantly induced IgM and IgG, while IgA was virtually absent, even at day 20 after vaccination. In contrast, although not significantly different from AZD1222, BNT162b2 elicited little IgM. There were though increased IgG and IgA titers in BNT162b2-vaccinated subjects and these differences reached statistical significance on day 20 for IgG and already on day 13 for IgA (Fig. 7A). When looking at response rates instead of Ig titers, there were significantly more IgM and significantly less IgA responders to AZD1222 compared to BNT162b2 as calculated via Fisher’s exact test (Additional file 1: Table S3). When testing for surrogate virus neutralization, we found that SARS-CoV-2 spike receptor binding domain neutralization was 1.6-fold higher for BNT162b2 (median 784 IU/mL) when compared to AZD1222 (median 482 IU/mL, *p* = 0.0175). Interestingly, Spearman rank correlation analyses indicated strong relationships between neutralizing antibodies and amounts of spike binding IgM (*r* = 0.6411, *p* < 0.001), IgG (0.9328, *p* < 0.001), and IgA (0.6952, *p* < 0.001), respectively. Collectively, disparate vaccine strategies for spike protein delivery impacted differently on the humoral immune response and shaped distinctive antibody isotype layouts as well as virus neutralization capacities after single doses of AZD1222 and BNT162b2.

## Discussion

We here compared early immune reactions to the primary vaccination against SARS-CoV-2 with either AZD1222 from AstraZeneca or BNT162b2 from BioNtech [6, 8]. While both vaccines elicited strong cellular and humoral responses, the individual impacts on the peripheral immune compartment were strikingly different. In detail, the adenoviral vector AZD1222 led to a transient yet profound leukopenia on day 2, involving significant reductions of B and T lymphocytes as well as granulocytes. As decreased leukocyte counts have previously been reported for both, regular adenoviral infections [21] and adenovirus-mediated gene therapeutic approaches [22, 23], we consider it likely that the leukopenia observed here can be attributed to the viral vector rather than the spike protein [24]. For example, Park and colleagues observed in an outbreak of adenoviral pneumonia among 191 otherwise healthy adults a high percentage of patients that exhibited febrile leukopenia [21]. Furthermore, Varnavski et al. observed a transient decrease of leukocyte counts in two separate studies in which a human adenoviral vector was applied in rhesus macaques and mice, respectively [22, 25].

Likewise, the AZD1222-induced changes among peripheral immune cell proportions were reminiscent of viral infections. For instance, short-term enrichments of pro-inflammatory monocytes have been observed in patients infected with dengue or human immunodeficiency virus, respectively [26, 27]. In detail, Kwissa and colleagues have shown that CD14^+^CD16^+^ monocytes were increased in both humans and non-human primates shortly after infection with the dengue virus and that these cells were able to stimulate the differentiation of plasmablasts [28]. Both the enrichment of CD4^+^ TEMRA and terminally differentiated cytotoxic CD8^+^ T cells have been associated with human cytomegalovirus [29–31]. In addition, the class-switched, late memory B cells that we observed after AZD1222 vaccination have previously been associated with an efficient control of viral infections [32, 33]. Together, our data suggest that it is the adenoviral vector rather than the encoded spike protein that elicits a significant immune cell response in the periphery.

By comparison, peripheral immune cell proportions appeared mostly unaffected by BNT162b2 administration, which corroborates the observation that this vaccine is globally well tolerated among first dose recipients [9]. Indeed, the BNT162b2 encoded mRNA bears an m1Ψ modification which attenuates innate immune sensing [34]. We therefore assume that the relatively small enrichment of peripheral plasmablasts after BNT162b2 vaccination, which can also be observed during COVID-19 [35], resulted from an adaptive response towards the SARS-CoV-2 spike protein whereas the larger enrichment observed with AZD1222 likely reflects a combined response towards the spike protein and the adenoviral vector. In order to verify this hypothesis, future studies should examine the proportions of peripheral plasmablasts and memory B cells that express a B cell receptor specific for either the spike protein or adenoviral antigens, respectively. In fact, a study by Ciabattini and colleagues demonstrated for subjects that were vaccinated with BNT162b2 spike-specific memory B cells that persisted in the circulation for at least 6 months after the second dose [36].

When comparing the specific antibody responses against the spike protein elicited by both vaccines, we found that the isotypes produced were significantly different with AZD1222 inducing primarily IgM and IgG compared to predominantly IgG and IgA by BNT162b2. Furthermore, we found that the amounts of plasma neutralizing antibodies were significantly elevated for BNT162b2 at day 20 after vaccination. We also demonstrated that receptor binding domain neutralization strongly correlated with spike binding IgM, IgG, and IgA which is in line with previous data [5, 37–39]. Even though we cannot yet predict whether this trend will be continued beyond the first 3 weeks after vaccination, a pronounced IgA response combined with a higher virus neutralization capacity following BNT162b2 vaccination may explain its superior effectiveness in preventing symptomatic COVID-19 after both infection with wild type SARS-CoV-2 and its variants [15, 17, 19, 40]. Indeed, Chan et al. demonstrated that, following vaccination with BNT162b2, IgA is detectable in nasal swabs and that plasma IgA levels correlate with their capacity for SARS-CoV-2 receptor binding inhibition which was also demonstrated for patients who recovered from COVID-19 [35, 41]. Optimizing existing vaccines might therefore also aim at alternative antigen delivery, e.g., towards mucosal sites, in order to support not only IgA production but also tissue-resident effector cells which will help contain viral loads at the nasopharyngeal entry sites [42, 43].

Optimizing vaccines may also aim at modifying potential bystander effects. When analyzing the spike protein-specific T cell responses, we observed that both vaccines elicited functional immune responses. Both vaccination regimes expanded effector cells to a comparable degree as documented by significant increases in activated CD25 and CD137 co-expressing CD4^+^ and CD8^+^ T lymphocytes as well as FasL expressing CD8^+^ cells upon re-challenge. However, when looking at intracellular cytokine production, AZD1222 induced a prominent type 1 helper T cell (Th1) response as illustrated by significant increases in IFNγ and TNFα, respectively. Because adenoviral vectors have previously been shown to facilitate strong cellular immunity towards the delivered antigen and drive the expansion of Th1 cells [6], we believe that this Th1 reaction towards the adenoviral vector exerted some bystander effect on the response against the spike protein. However, an inordinate Th1 response may foster a cytokine layout that is hardly supportive of class-switch recombination towards IgA [44]. On the other hand, we assessed neither IL-5 nor transforming growth factor (TGF)β and are therefore are not yet able to verify whether BNT162b2 indeed induced more IgA promoting cytokines.

Of note, our ELISPOT experiments addressing spike protein-specific Th1 cells before and after vaccination found an even stronger induction of IFNγ-producing T helper cells among BNT162b2 compared to AZD1222 vaccinees. Even though these results seemingly contradict the intracellular cytokine readout of stimulated vs unstimulated day 20 T cells, they show that both vaccines induced Th1 responses. In addition, we found a reduction of IL-2 and IL-10 co-producing CD4^+^ and CD8^+^ T cells upon in vitro re-stimulation which however only reached significance in the case of AZD1222 vaccination. IL-10 is a hallmark of regulatory T cells and exerts anti-inflammatory effects via suppressing not only effector T cells, but also antigen presentation and the secretion of inflammatory cytokines by APCs [45–47]. We like to speculate that this significant reduction of IL-10 expression is not restricted to in vitro re-challenge but may also occur after booster immunization and perpetuate a Th1 response that impedes an IgA promoting cytokine milieu.

This study has a few limitations, among them the small sample sizes. Furthermore, we did not account for any possible underlying conditions nor did we document the general health status of the study participants that might influence the variance within and between both vaccine groups. Nonetheless, our results depict for both vaccines significantly disparate effects on the peripheral immune layout and on the regulation of T cell effector molecules and we assume that these differences were generated by the different strategies for spike antigen delivery. Another limitation is the lack of virus neutralization data using SARS-CoV-2. However, recent data demonstrated that readouts from surrogate virus neutralization robustly correlate with conventional virus neutralization and are therefore a suitable measure for humoral protection from infection [48]. In summary, we consider the description of disparate vaccine effects on the immediate immune response the strength of our study and we believe that our results will be of use for further optimization of vaccination strategies.

## Conclusions

We here show that, after a single dose, the SARS-CoV-2 vaccines AZD1222 and BNT162b2 impact differently on the peripheral immune compartment. Although both vaccines elicited the induction of spike-specific effector cells and spike binding antibodies, the different compilation of these immunological features suggests that the strategy for spike delivery impacts on how and to what extent immune priming against the main SARS-CoV-2 antigen proceeds. We propose that the induction of higher quantities of IgA might be associated with superior protection against breakthrough infections after booster injections. Conclusively, additional investigations are needed that further our understanding about the immunization mechanisms that lead to a favorable humoral and cellular layout that is effective against COVID-19.

## Methods

### Study design

This explorative study was designed to compare the immediate immune response towards a single-dose vaccination with either AZD1222 or BNT162b2. For that, study participants were recruited from the coordination center for clinical studies at the Rostock University Medical Center. Individuals with a study-independent appointment at a vaccination center were eligible to participate. No other specific inclusion criteria were set. Apart from their age and sex, no other personal data (i.e., underlying medical conditions) were documented. Blood samples were obtained by consecutive venipuncture immediately before vaccination (day 0) and on days 2, 6, 13, and 20 thereafter. Participants that were positive for SARS-CoV-2 spike protein binding IgG were excluded from further analyses. PBMCs were isolated from anti-coagulated blood by density gradient centrifugation using Ficoll-Paque^TM^ PLUS to the manufacturer’s instructions (Cytiva). PBMCs were subsequently suspended in fetal calf serum (Thermo Fisher Scientific) containing 10% dimethyl sulfoxide (Sigma-Aldrich) and were frozen at −70 °C until further use. Plasma samples were obtained by centrifugation of anti-coagulated blood and were frozen afterwards. This study was approved by the ethics committee of the Rostock University Medical Center under file number A 2020-0086. Written informed consent was provided by all participants.

### Flow cytometric analyses on surface markers

For the analysis of surface expression markers, 100 μL of anti-coagulated whole blood was used. In order to reduce unspecific antibody-conjugate binding, 10 μL FCS, 5 μL True-Stain Monocyte Blocker^TM^, and 5 μL anti-Fc receptor TruStain FcX^TM^ (BioLegend) were added and incubated for 15 min on ice. The following amounts of antibody:fluorophore combinations were used: 0.25 μg CD127:APC/R700 (clone HIL-7R-M21), 1 μg CD147:BV421 (TRA-1-85), 0.5 μg CD45RO:BV480 (UCHL1, BD Biosciences), 1 μg CD11b:PerCP/Cy5.5 (ICRF44), 0.8 μg CD11c:BV785 (3.9), 0.56 μg CD14:BV510 (63D3), 0.13 μg CD16:BV650 (3G8), 0.06 μg CD19:APC/Fire810 (HIB19), 0.13 μg CD20:SparkNIR685 (2H7), 0.5 μg CD27:BV605 (O323), 0.25 μg CD3:SparkBlue550 (SK7), 0.25 μg CD304:AlexFluor647 (12C2), 0.03 μg CD4:BV750 (SK3), 0.5 μg CD45RA:APC/Fire750 (HI100), 0.13 μg CD56:BV711 (5.1.H11), 0.13 μg CD8:BV570 (RPA-T8), 0.5 μg CD95:PE/Cy5 (DX2), 0.13 IgD:PE/Dazzle594 (IA6-2), 0.13 μg PD-1:APC (A17188B, BioLegend), 0.06 μg CD38:PerCP/eFluor710 (HB7, Thermo Fisher Scientific), 0.06 μg CD177:PE/Vio770 (REA258), and 0.05 μg CD25:PE (REA570, Miltenyi Biotec).

Antibodies were incubated for 15 min on ice in the dark. Subsequently, Apotracker^TM^ Green (BioLegend) was added according to the manufacturer’s instruction without washing, followed by incubation for 30 min on ice. In order to lyse erythrocytes, 2.2 mL Fixative-Free Lysing Solution (Thermo Fisher Scientific) was added and incubated for 20 min at room temperature. Subsequently, 0.03 μg 4′,6-diamidino-2-phenylindole (DAPI, BioLegend) was added as a live/dead discriminator and incubated for 5 min. Finally, data acquisition was performed on the Cytek® Aurora flow cytometer running on the SpectroFlo Software version 2.2.0.3 (Cytek Biosciences). Analysis of flow cytometry data was done using FlowJo software version 10.7 (FlowJo). The gating scheme is shown in Additional file 1: Fig. S6. Dimension reduction of down-sampled (5 × 10^4^ live cells per sample) and concatenated data sets was performed using the FlowJo plugin for the algorithm UMAP [49].

### Interferon gamma ELISPOT

PBMCs from day 0 and day 20 were thawed, centrifuged, and suspended in a complete RPMI medium containing 10% FCS, 1% penicillin/streptomycin, 2 mM l-glutamine (Thermo Fisher Scientific), 10 mM HEPES, and 1 mM sodium pyruvate (PAN-Biotech). Cell counts were determined cytometrically on the Cytek® Aurora (Cytek Biosciences) using DAPI (BioLegend) as a live/dead discriminator. Five hundred thousand PBMCs were pipetted into a 96-well U-bottom plate and centrifuged for 5 min at 4 °C and 400×*g*. Subsequently, supernatants were removed by carefully blotting the plate on a paper tissue. Cells were then suspended in a 36-μL complete RPMI medium containing 0.2 μg of the SARS-CoV-2 trimeric spike protein (R&D Systems). Afterwards, PBMCs were transferred into a 96-well ELISPOT assay plate coated with capture antibodies specific for human IFNγ (R&D Systems). After incubating the cells for 30 min at 37 °C, 164 μL of complete RPMI medium was added to all wells followed by 24-h incubation at 37 °C in a CO_2_ incubator (BINDER). The ELISPOT assay was then performed according to the manufacturer’s guidelines. The numbers of IFNγ-producing cells were determined by automated counting using the ImmunoSpot® analyzer running on the ImmunoSpot® Software version 5.0.9.15 (CTL Europe). The counts of IFNγ-positive cells were normalized to their respective paired sample from d0.

### T cell re-stimulation and intracellular cytokine staining assay

For the establishment of T cell re-stimulation and intracellular cytokine staining assays, blood was collected from six vaccinated subjects 2 to 29 weeks after the last dose and from one patient who had recovered from COVID-19 presumably 24 weeks prior to venipuncture. In detail, one subject was vaccinated with a single dose of Ad26.COV2.S (Johnson & Johnson), three individuals received two doses of BNT162b2 (BioNTech), one received one dose of BNT162b2 and one received one dose of AZD1222 (AstraZeneca) followed by one dose of BNT162b2. PBMCs were isolated and frozen until further use in the same fashion as described above. For assaying the vaccinated study participants, day 20 PBMCs were used. Upon thawing, PBMCs were counted as described above and aliquots of 0.8 million were seeded into single wells of 96-well U-bottom plates. Every sample was stimulated at least in duplicates. After centrifugation, cells were re-suspended and stimulated in a total volume of 36-μL complete RPMI medium with either 1 μg of the BNT162b2 vaccine or 0.2 μg of the SARS-CoV-2 trimeric spike protein or left without any stimulation. Pooled leftovers of the BNT162b2 vaccine, which are not allowed to be used for administration in Germany, were kindly provided by staff members of the Rostock vaccination center. Re-stimulation with BNT162b2 was preferred over re-stimulation with AZD1222 because we expected an activation of T cells that are reactive against adenoviral antigens from the vector which would obscure the isolated response towards the spike protein primarily in the group of participants that were vaccinated with AZD1222. Phorbol 12-myristate-13-acetate (PMA, 10 ng/ mL) and Ionomycin (1 μg/mL) stimulated samples were processed in parallel as positive controls. After adding 164 μL of complete RPMI medium, cells were incubated for 20 h under 5% CO_2_ atmosphere at 37 °C. One microgram of Brefeldin A (Sigma-Aldrich) was added thereafter followed by incubation for another 4 h.

Successive incubation steps were performed in the dark. Duplicate samples were pooled, washed in PBS (Thermo Fisher Scientific), suspended in PBS containing 2000-fold diluted ZombieNIR dye (BioLegend), and incubated for 20 min at room temperature. Thereafter, cells were washed and suspended in autoMACS® Running Buffer (RB, Miltenyi Biotec). Subsequently, unspecific antibody-conjugate binding sites were blocked by adding FCS, True-Stain Monocyte Blocker^TM^, and anti-Fc receptor TruStain FcX^TM^ (BioLegend) for 10 min at room temperature. Surface antigens were stained by incubating the cells with the following antibody:fluorophore combinations: 1.25 μg CD3:FITC (clone UCHT1), 0.02 μg CD4:BV750 (SK3), 0.06 μg CD8:BV570 (RPA-T8), 0.5 μg Fas-L:PE (NOK1), 1 μg CD25:APC (BC96, BioLegend), 1.25 μL CD127:APC/R700 (HIL-7R-M21), and 0.25 μg CD137:BV480 (4B4-1, BD Biosciences) for 15 min at room temperature. Cells were subsequently washed in RB, suspended in 100 μL Fixation Buffer (BioLegend), and incubated at room temperature for 20 min. Cells were washed twice and then suspended in Intracellular Staining Permeabilization Wash Buffer (BioLegend). After blocking unspecific binding sites as described above, 0.5 μg Granzym B:AlexaFluor647 (clone 6B11), 2.5 μg IFNγ:PerCP/Cy5.5 (4S.B3), 0.63 μg IL-2:BV650 (MQ1-17H12), 0.3 μg IL-4:PE/Dazzle594 (MP4-25D2), 1 μg IL-10:BV421 (JES3-907, BioLegend), and 0.13 μg TNFα:PE/Cy7 (Mab11, Thermo Fisher Scientific) were added and incubated for 30 min at room temperature. Finally, cells were washed twice in RB. Data acquisition and analysis of expression data was performed as described above. The gating scheme for the intracellular cytokine staining assay is shown in Additional file 1: Fig. S7.

### SARS-CoV-2 trimeric spike-specific antibodies

Plasma samples from all time points were thawed on ice and centrifuged at 10,000×*g* in order to remove precipitates. For the detection of SARS-CoV-2 trimeric spike protein-specific IgG and IgA levels, plasma was diluted 101-fold. The ELISA for these isotypes was conducted after the manufacturer’s specifications (EUROIMMUN). In order to determine IgM levels, plasma samples were diluted 1000-fold and the ELISA was performed to the manufacturer’s instructions (Thermo Fisher Scientific). The absorbance was detected at 450 nm (A450) on the Infinite® 200 automated plate reader (Tecan Group). Absorbance readouts were normalized to calibrator values and were reported as arbitrary units. According to the manufacturer’s guidelines, calibrated sample values between 0.8 and 1.1 were considered borderline and above 1.1 were considered clearly positive. Samples from individuals with arbitrary unit values of less than 0.8 were considered non-responders.

### Surrogate virus neutralization

Plasma samples were processed like described above. For standardization, we ran plasma samples from the World Health Organization (WHO) Reference Panel in parallel [50]. For the determination of antibody neutralization, we utilized the SARS-CoV-2 Surrogate Neutralization Test (sVNT) Kit (GenScript) to the manufacturer’s guidelines. In brief, plasma samples and standards were diluted 10-fold and were incubated with a horseradish peroxidase-conjugated SARS-CoV-2 receptor binding domain for 30 min at 37 °C. Subsequently, sample mixtures were pipetted onto an ELISA plate coated with the ACE2 receptor. After incubation at 37 °C for 15 min, wells were washed four times and color reaction was initiated by the addition of TMB substrate. Absorbances at 450 nm were detected on the automated plate reader after the reaction was quenched. A450 from a series of WHO standards with known neutralization capacity were fitted with an exponential model by the formula: International Units per mL (IU/mL) = a × exp(b × A450). IU/mL for plasma samples were computed with this model.

### Statistical analysis

Data analyses were performed using R (version 3.5.1) and InStat version 3.10 (GraphPad Software). All data were analyzed by two-sided testing. Data sets were evaluated for Gaussian distribution using the Kolmogorov-Smirnov test. Under the assumption of normally distributed sample data, multiple independent groups were compared by one-way analysis of variance (ANOVA) followed by post hoc pairwise comparisons using the Tukey-Kramer test. Data, which did not follow Gaussian distribution, was compared by the Kruskal-Wallis one-way analysis of variance combined with Dunn’s test for multiple comparisons. The one-sample *t*-test was performed to compare single group means to a hypothetical value. Differences between dependent samples were assessed by the paired *t*-test given normal distribution and by the Wilcoxon signed rank test in case of deviation from Gaussian distribution. Correlation analyses were performed using the Spearman rank method. A *p*-value of less than 0.05 was considered statistically significant. Data visualization was performed with SigmaPlot version 13.0 (Systat Software).

## Supplementary Information

**Additional file 1.** Supplementary figures and tables

## Data Availability

All data generated or analyzed during this study are included in this published article and its supplementary information file.

## Abbreviations

ACE2: Angiotensin-converting enzyme 2
APC: Antigen-presenting cell
COVID-19: Coronavirus disease 2019
DAPI: 4’,6-Diamidino-2-phenylindole
ELISA: Enzyme-linked immunosorbent assay
ELISPOT: Enzyme-linked immune absorbent spot [assay]
EMA: European Medicines Agency
HLA: Human leukocyte antigen
IFNγ: Interferon γ
IL: Interleukin
m1ψ: N1-methyl-pseudouridine
PBMC: Peripheral blood mononuclear cell
PMA: Phorbol 12-myristate-13-acetate
RB: Running Buffer
SARS-CoV-2: Severe acute respiratory syndrome coronavirus 2
TEMRA: Effector memory T cells re-expressing RA
TGFβ: Transforming growth factor β
Th1: Type 1 helper T cell
TNFα: Tumor necrosis factor α
UMAP: Uniform manifold approximation and projection
WHO: World Health Organization
