# Supplementary material for: Single-dose SARS-CoV-2 vaccinations with either BNT162b2 or AZD1222 induce disparate Th1 responses and IgA production
Source: BMC Med. 2022 Jan 19;20:29. doi: 10.1186/s12916-022-02240-4 (PMC8766223; doi:10.1186/s12916-022-02240-4)
Supplement: Supplementary file 1 — Additional file 1. Supplementary figures and tables [file 12916_2022_2240_MOESM1_ESM.docx]

Additional File 1

Supplementary Information for

**Single-Dose SARS-CoV-2 Vaccinations With either BNT162b2 or AZD1222 Induce Disparate Th1 Responses and IgA Production**

Michael Müller, Johann Volzke *et al.*

Corresponding author. Email: Johann Volzke, johann.volzke@med.uni-rostock.de

**The file includes:**

Figs. S1 to S7

Tables S1 to S3


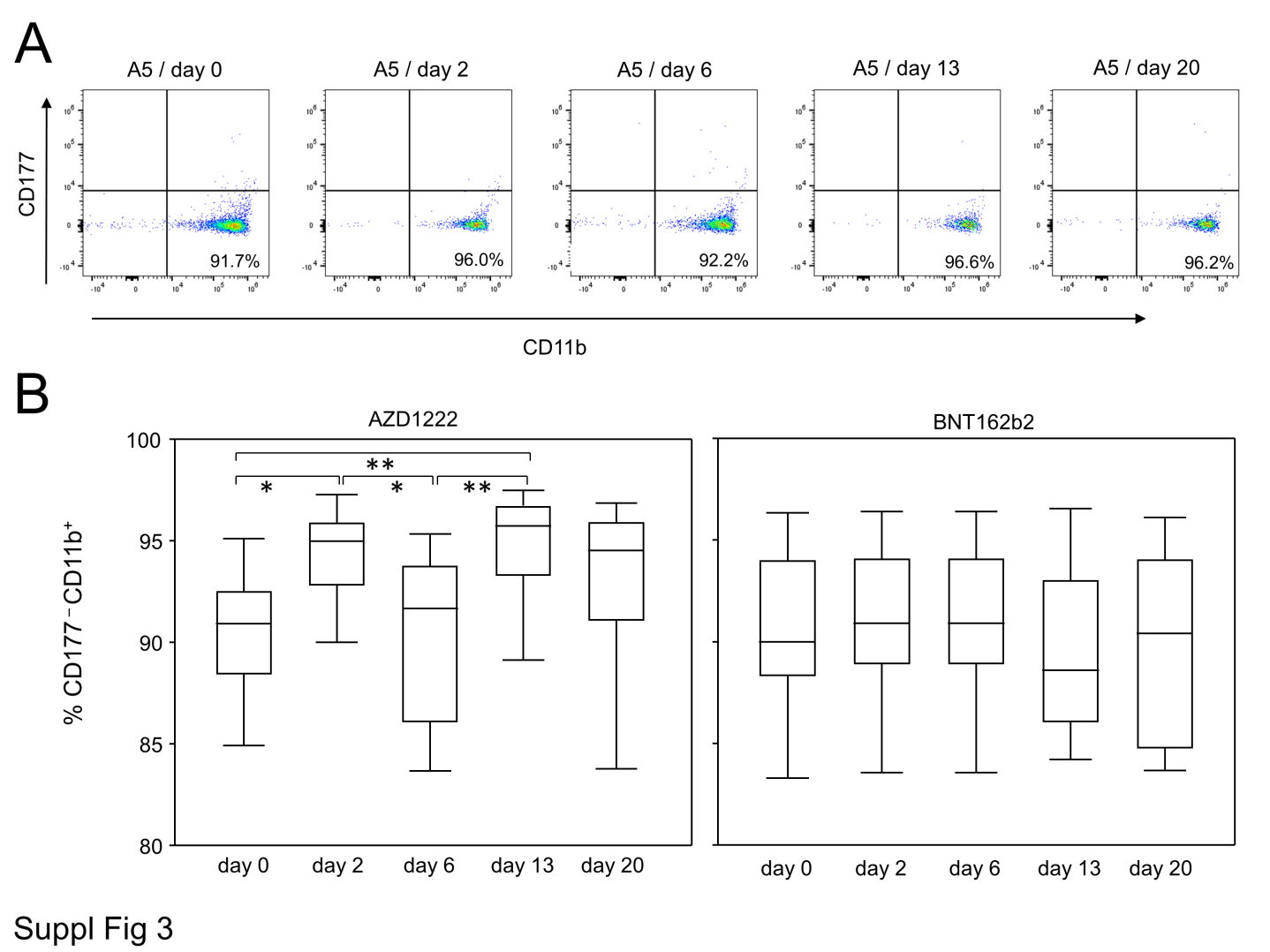
 Figure S1. **Vaccination with AZD1222, unlike BNT162b2, led to an intermittent increase in CD14^+^CD16^–^CD11b^+^CD177^–^ granulocytes.** (**A**) Pseudocolor plots for the expression of CD11b and CD177 on SSC^hi^CD14^+^CD16^–^ granulocytes are representative for the AZD1222 vaccination group. (**B**) Proportions of CD177^–^CD11b^+^ granulocytes after vaccination with AZD1222 (n = 18, left panel) or BNT162b2 (n = 18, right panel). p-values resulting from Kruskal-Wallis and Dunn´s multiple comparisons tests were 0.0004 for AZD1222 and 0.6760 for BNT162b2 analyses, respectively. *p < 0.05, **p < 0.01


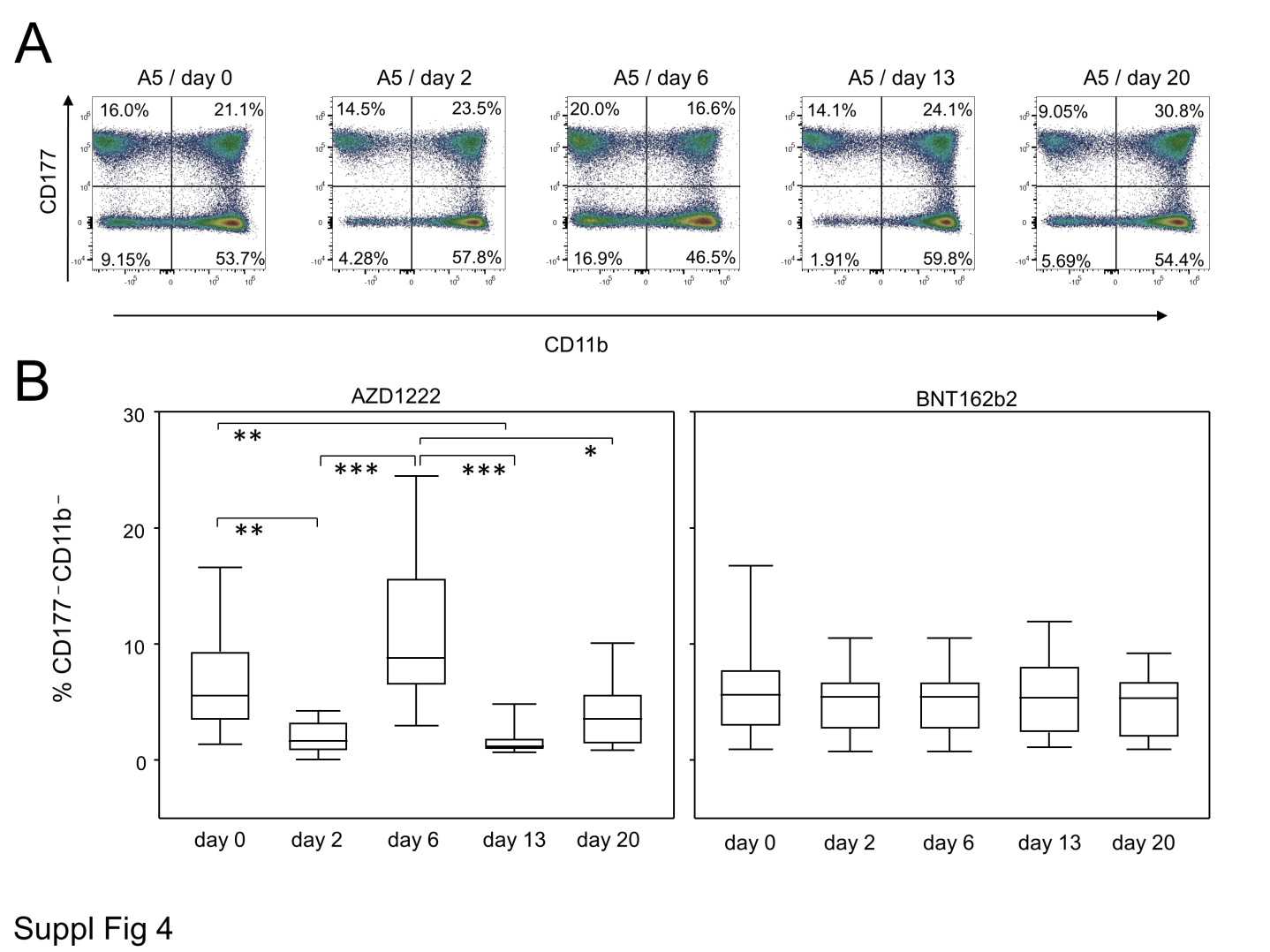


Figure S2. **CD14^+^CD16^+^CD11b^–^CD177^–^ granulocytes were decreased after vaccination with AZD1222 only.** (**A**) Pseudocolor plots for the expression of CD11b and CD177 on SSC^hi^CD14^+^CD16^+^ granulocytes are representative for the AZD1222 vaccination group. (**B**) Proportions of CD177^–^CD11b^–^ granulocytes after vaccination with AZD1222 (n = 18, left panel) or BNT162b2 (n = 18, right panel). FACS analyses were gated on CD14^+^CD16^+^ granulocytes. p-values resulting from Kruskal-Wallis and Dunn´s multiple comparisons tests were < 0.0001 for AZD1222 and 0.9152 for BNT162b2 analyses, respectively. *p < 0.05, **p < 0.01, ***p < 0.001

**
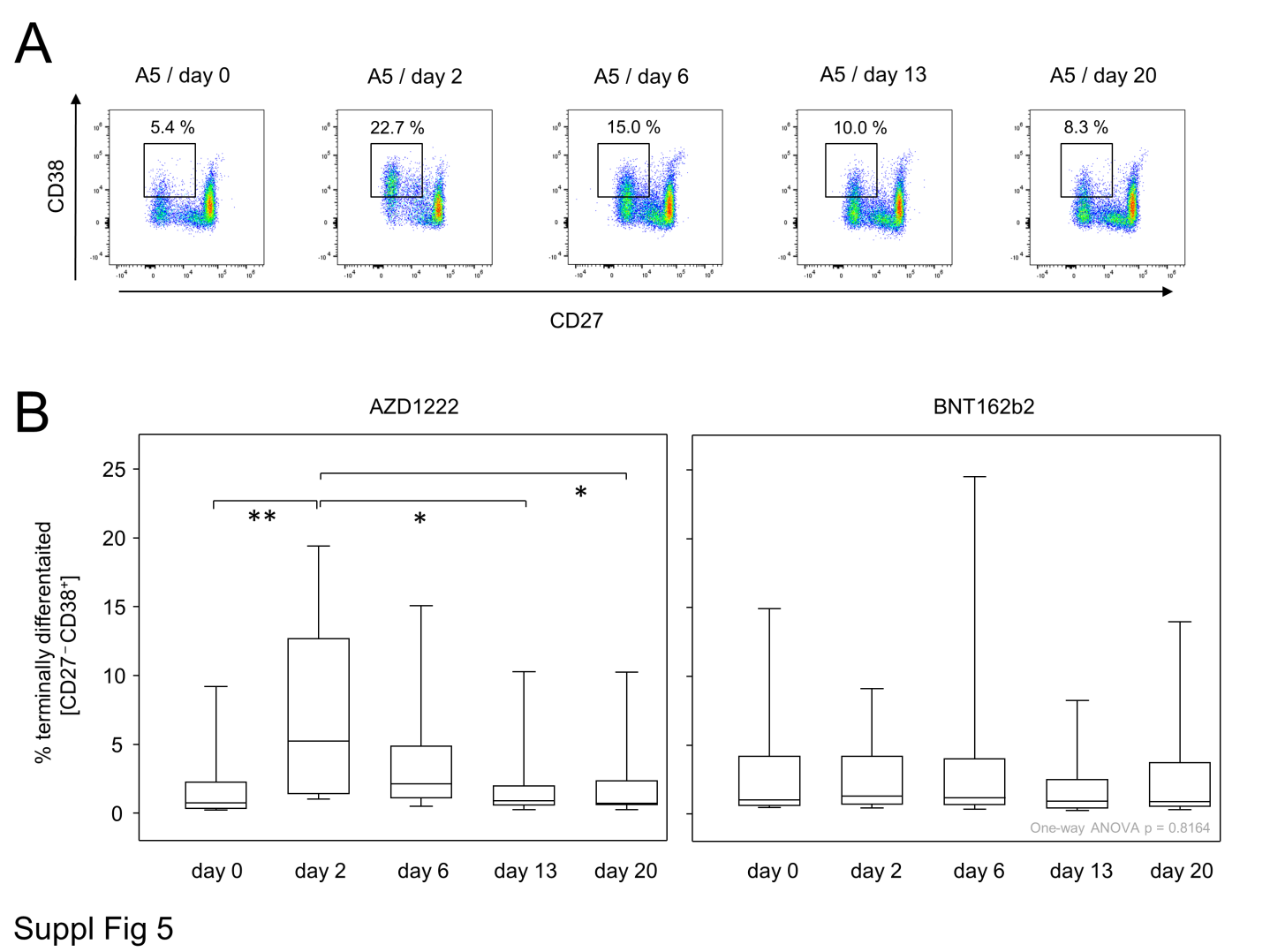
**

Figure S3. **AZD1222 vaccination induced the transient increase of CD27^–^CD38^+^ terminally differentiated CD8^+^ T cells.** (**A**) Pseudocolor plots for the expression of CD27 and CD38 on CD8^+^ T cells are representative for the AZD1222 vaccination group. (**B**) Proportions of CD8^+^CD27^–^CD38^+^ T cells after vaccination with AZD1222 (n = 18, left panel) or BNT162b2 (n = 18, right panel). All FACS analyses were on CD8^+^ T cells. p-values resulting from Kruskal-Wallis and Dunn´s multiple comparisons tests were 0.0009 for AZD1222 and 0.7905 for BNT162b2 analyses, respectively. *p < 0.05, **p < 0.01

**
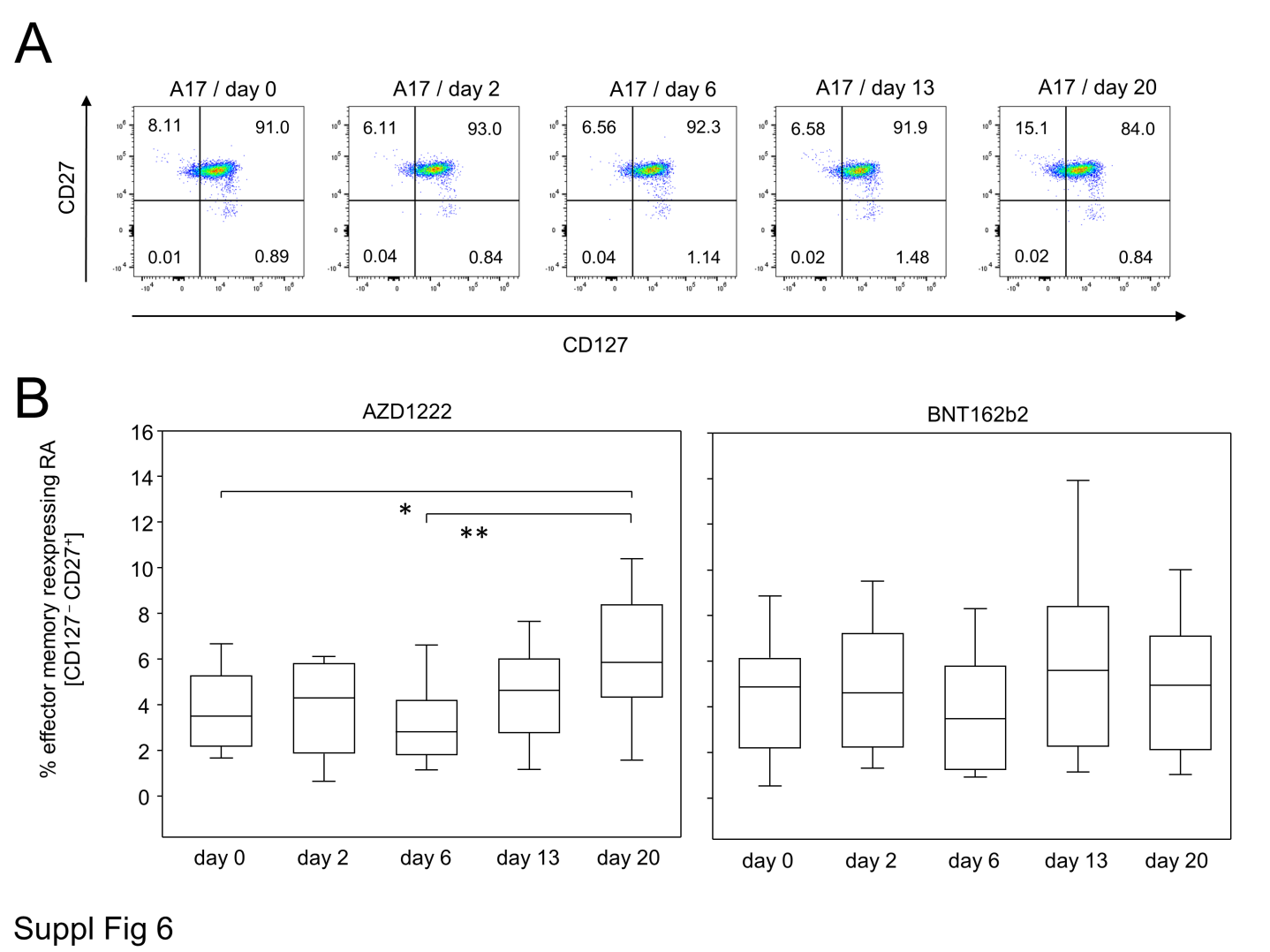
**

Figure S4. **CD4^+^ effector memory T cells re-expressing RA were increased towards the end of the observation period after AZD1222 vaccination.** (**A**) Pseudocolor plots for the expression of CD127 and CD27 on CD4^+^ T cells are representative for the AZD1222 vaccination group. (**B**) Proportions of CD4^+^CD127^+^CD27^+^ T cells after vaccination with AZD1222 (n = 18, left panel) or BNT162b2 (n = 18, right panel). All FACS analyses were on CD4^+^ T cells. p-values resulting from one-way ANOVA and Tukey-Kramer multiple comparisons tests were 0.0063 for AZD1222 and 0.4955 for BNT162b2 analyses, respectively. *p < 0.05, **p < 0.01


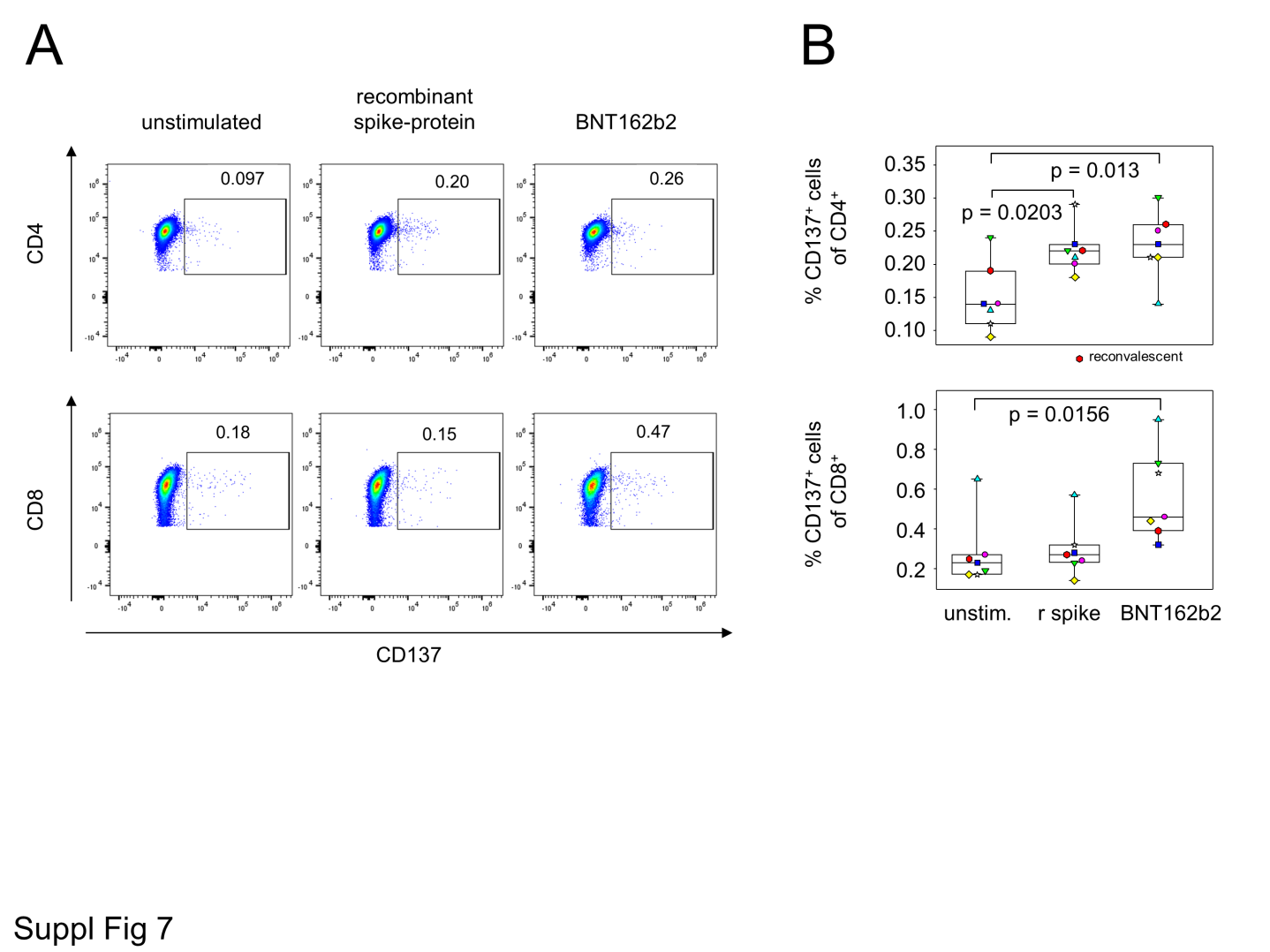


Figure S5. **Establishment of a protocol for the re-stimulation of SARS-CoV-2 spike protein specific immune cells.** PBMCs were isolated from fully vaccinated (n = 6) and COVID-19 convalescent (n = 1) blood donors and were in vitro re-stimulated with either recombinant spike protein (r spike) or the spike protein encoding mRNA (BNT162b2). (**A**) Representative pseudocolor plots showing the expression of CD137 on CD4^+^ (upper panel) or CD8^+^ T cells (lower panel) exemplifies activation. (**B**) Quantitative data for different stimulation regimen show that recombinant spike protein induced the enrichment of activated CD4^+^ T cells, whereas spike protein encoding mRNA was able to increase the proportions of both activated CD4^+^ and CD8^+^ T cells. Red dots indicate the patient that recovered from COVID-19.

**
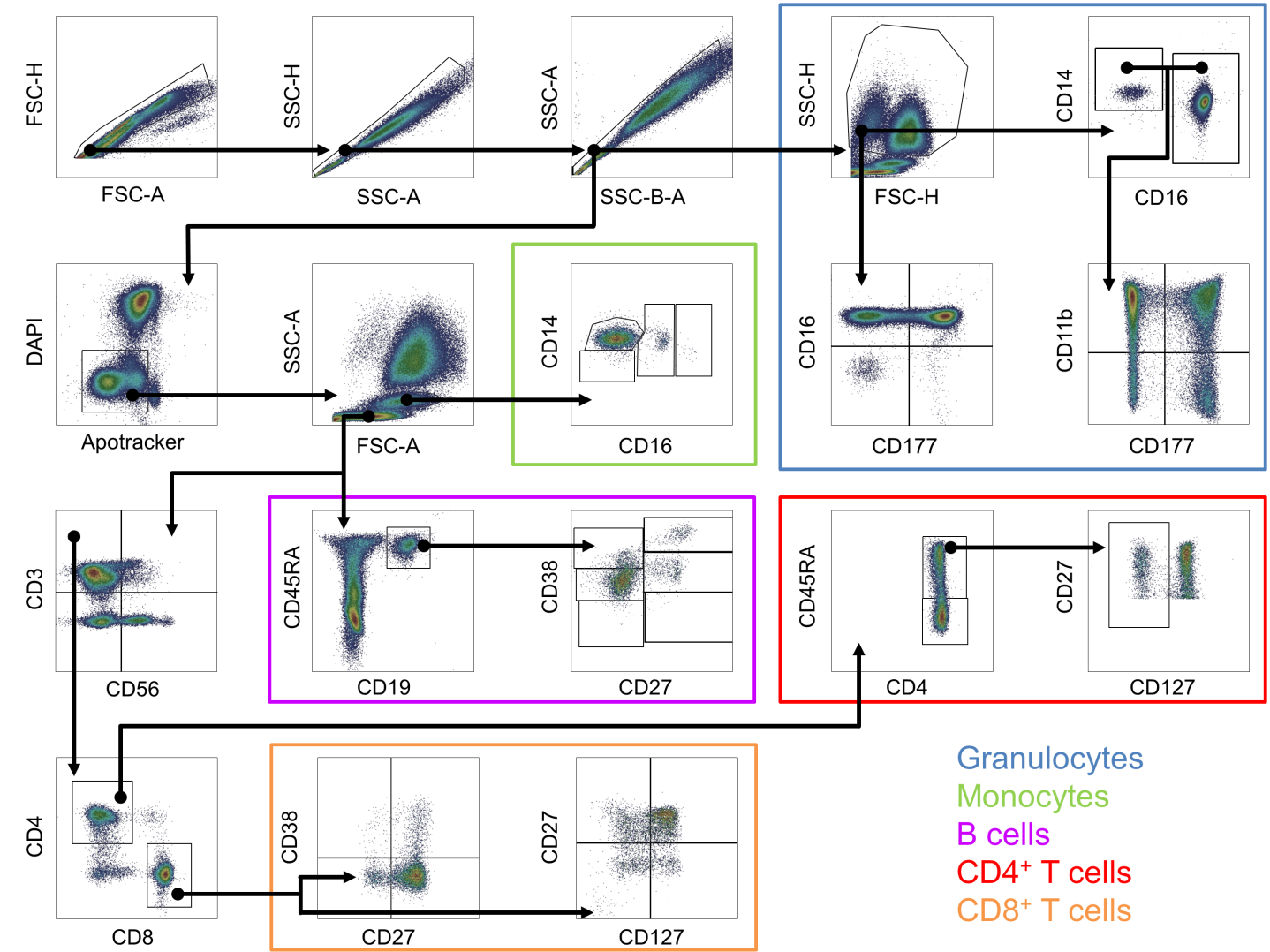
**

Figure S6. **Gating scheme for the 24-colour immune-phenotyping of peripheral whole blood cells.** Arrows indicate the hierarchical gating steps.


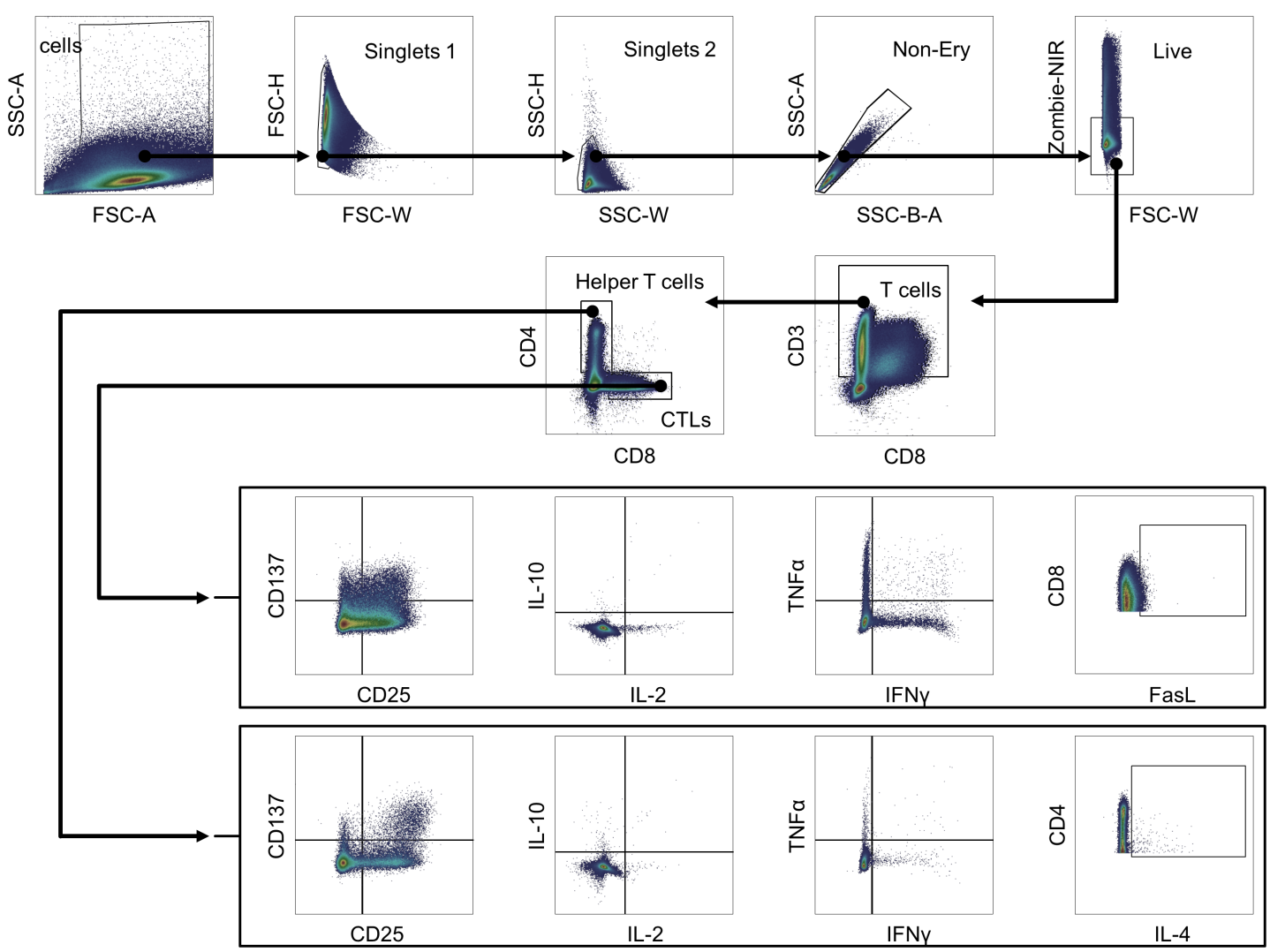
 Figure S7. **Gating scheme for the intracellular cytokine staining assay, here exemplified for PMA/Ionomycin stimulated PBMC.** Expression of the activation markers CD25, CD137 as well as the cytokines IL-2, IL-10, IFNγ and TNFα were analyzed on CD4^+^ and CD8^+^ cells, respectively. IL-4 production was analyzed only on CD4^+^ cells while FasL expression was examined for CD8^+^ cells only. Arrows indicate the hierarchical gating steps.

Table S1. Immune status during early AZD1222 vaccination response.

|  | Day 0 (n=18)  cells/µL [median]  (IQR) | Day 2 (n=15)  cells / µL [median]  (IQR) | Day 6 (n=17)  cells / µL [median]  (IQR) | Day 13 (n=16)  cells / µL [median]  (IQR) | Day 20 (n=16)  cells / µL [median]  (IQR) | p-value  (Kruskal-Wallis) |
| --- | --- | --- | --- | --- | --- | --- |
| granulocytes | 2394  (1842-3093) | 632  (479-1101) | 2004  (1618-2232) | 2086  (1427-2862) | 2107  (1355-2850) | < 0.0001 |
| monocytes | 369  (253-448) | 269  (226-311) | 278  (252-412) | 252  (189-349) | 297  (174-372) | 0.3110 |
| lymphocytes | 1846  (1788-2658) | 1014  (698-1262) | 1803  (1560-2689) | 1925  (1585-2687) | 1827  (1439-2338) | 0.0009 |
| B cells | 216  (140-287) | 79  (57-160) | 162  (126-261) | 246  (187-409) | 151  (122-278) | 0.0019 |
| T cells | 1102  (873-1524) | 560  (416-695) | 1114  (969-1419) | 1178  (893-1416) | 1005  (750-1230) | 0.0004 |
| CD4+ T cells | 684  (521-1092) | 333  (262-466) | 734  (596-936) | 799  (584-954) | 637  (494-822) | 0.0026 |
| CD8+ T cells | 325  (248-358) | 149  (89-185) | 299  (252-337) | 291  (214-351) | 252  (209-313) | < 0.0001 |

Table S2. Immune status during early BNT162b2 vaccination response.

|  | Day 0 (n=18)  cells/µL [median]  (IQR) | Day 2 (n=17)  cells / µL [median]  (IQR) | Day 6 (n=18)  cells / µL [median]  (IQR) | Day 13 (n=16)  cells / µL [median]  (IQR) | Day 20 (n=17)  cells / µL [median]  (IQR) | p-value  (Kruskal-Wallis^#^)  (one-way ANOVA*) |
| --- | --- | --- | --- | --- | --- | --- |
| granulocytes | 1330  (1132-2100) | 1240  (1043-1683) | 1571  (1239-2246) | 1173  (831-1836) | 1646  (1098-2221) | 0.3834^#^ |
| monocytes | 345  (215-420) | 329  (251-391) | 303  (253-371) | 331  (257-389) | 307  (216-407) | 0.9404* |
| lymphocytes | 1829  (1332-2076) | 1816  (1335-2023) | 1756  (1603-2097) | 1746  (1441-2674) | 1704  (1492-2401) | 0.7708* |
| B cells | 158  (125-260) | 147  (132-210) | 233  (136-388) | 178  (120-292) | 229  (125-307) | 0.0885* |
| T cells | 945  (726-1194) | 799  (705-1104) | 946  (641-1053) | 986  (648-1219) | 1046  (761-1409) | 0.4438^#^ |
| CD4+ T cells | 558  (490-617) | 511  (409-578) | 543  (370-606) | 589  (386-754) | 623  (513-849) | 0.3272^#^ |
| CD8+ T cells | 249  (183-381) | 236  (167-345) | 241  (197-361) | 256  (196-424) | 302  (230-457) | 0.6468^#^ |

Table S3. Responders to vaccines.

|  | IgM [% responders] | | IgG [% responders] | | IgA [% responders] | |  |
| --- | --- | --- | --- | --- | --- | --- | --- |
| vaccine | AZD1222 | BNT162b2 | AZD1222 | BNT162b2 | AZD1222 | BNT162b2 | |
| day 0 | 0 | 0 | 5.3 | 0 | 0 | 0 | |
| day 2 | 0 | 0 | 0 | 0 | 0 | 0 | |
| day 6 | 0 | 0 | 11.1 | 0 | 0 | 5.6 | |
| day 13 | 52.9 | 26.7 | 47.1 | 86.7 | 11.8 | 100 | |
| day 20 | 52.9 | 47.1 | 82.4 | 100 | 23.5 | 100 | |
| Fisher´s exact test [p value] | 0.033 | | 0.55 | | 0.040 | |  |
